# Quest for the Nitrogen-Metabolic Versatility of Microorganisms in Soil and Marine Ecosystems

**DOI:** 10.3390/microorganisms12071283

**Published:** 2024-06-25

**Authors:** Yongpeng Zhao, Xia Zhu-Barker, Kai Cai, Shuling Wang, Alan L. Wright, Xianjun Jiang

**Affiliations:** 1 College of Resources and Environment, Southwest University, 2 Tiansheng Road, Beibei, Chongqing 400715, China; 2 Department of Soil Science, University of Wisconsin-Madison, Madison, WI 53709, USA; 3 Indian River Research & Education Center, University of Florida-IFAS, Fort Pierce, FL 34945, USA

**Keywords:** metabolic versatility, N-transforming microbes, functional traits, metagenomics, N-cycling

## Abstract

Whether nitrogen (N)-metabolic versatility is a common trait of N-transforming microbes or if it only occurs in a few species is still unknown. We collected 83 soil samples from six soil types across China, retrieved 19 publicly available metagenomic marine sample data, and analyzed the functional traits of N-transforming microorganisms using metagenomic sequencing. More than 38% and 35% of N-transforming species in soil and marine ecosystems, respectively, encoded two or more N-pathways, although N-transforming species differed greatly between them. Furthermore, in both soil and marine ecosystems, more than 80% of nitrifying and N-fixing microorganisms at the species level were N-metabolic versatile. This study reveals that N-metabolic versatility is a common trait of N-transforming microbes, which could expand our understanding of the functional traits of drivers of nitrogen biogeochemistry.

## 1. Introduction

Nitrogen (N) is an essential element for all living organisms and is required for the synthesis of proteins and nucleic acids. Global N cycling is mainly dependent on microbes mediating multiple N transformation processes. Studies on N-cycling microorganisms mainly focused on taxonomic information [1,2,3,4], with less research on their functional traits. Microbial functional traits are features associated with organismal fitness or performance [5,6]. Microbial functional traits can be classified as phenotypic traits and genotypic traits, with phenotypic traits delineating morpho-physio-phenological characteristics and environmental preference and genotypic traits referring to functional genes or metabolic pathways of organisms [7]. Genotypic traits are increasingly recognized as the essential link between microbial communities and ecosystem functioning [8,9]. Therefore, deeper knowledge of microbial N-functional traits could lead to a better prediction of the N biogeochemistry in ecosystems.

The microbial N cycle can be broadly divided into eight N transformation pathways, namely ammonia assimilation, assimilatory nitrate reduction, dissimilatory nitrate to nitrite, dissimilatory nitrite to ammonia, denitrification, nitrification, nitrogen fixation, and anammox [10,11], and microorganisms are conventionally thought to involve in only one N-pathway [12]. Nevertheless, a *Pseudomonas* sp. type strain H8, a strain belonging to ‘nitrogen fixers’, was found to fix N_2_ gas and denitrify nitrate simultaneously [13]. Additionally, another known N-fixing rhizobia, *Bradyrhizobium japonicum*, is capable of encoding denitrifying gene (*nosZ*) to mitigate N_2_O emissions [14]. A recent study reported that a ubiquitous autotrophic nitrite-oxidizing bacteria (NOB) that is generally considered metabolically restricted to oxidize nitrite, *Nitrospira moscoviensis*, can actually convert urea to ammonia, which indicates that NOBs are unexpectedly flexible and can contribute to N-cycling beyond nitrite oxidation [15]. These studies suggested that certain microbes can be N-metabolically versatile. Genomic data collected in recent years also revealed the possibility of microorganisms being N-metabolically versatile [10]. However, it remains unclear whether N-metabolic versatility is a common trait of N-transforming microbes or does it only occur in a few select microbes. To address the gap, a shotgun metagenomic approach was used to characterize the N-functional traits of N-cycling species and 83 soil samples under different land use practices were collected from six soil types across China to explore the prevalence of N-metabolically versatility. The metagenomic data of marine microorganisms were also analyzed to verify the prevalence.

## 2. Materials and Methods

### 2.1. Study Sites and Sample Collection

The soils under different land uses (i.e., paddy, upland, forest, and fallow) were sampled from thirty sites across China, and a total of eighty-three samples were collected, including nineteen black soils (Phaeozems, FAO), nine fluvo-aquic soils (Fluvisols, FAO), nine yellow soils (Eumorthic Anthrosols, FAO), seventeen purple soils (Regosols, FAO), eight yellow-brown soils (Albic Luvisol), and twenty-one red soils (Ferralic Cambisol, FAO). The information of sampling sites and selected soil properties are shown in Table S1.

Soil samples were collected between May and October 2019 immediately after the removal of aboveground biomass. At each site, three replicated plots (4 m × 5 m) were randomly established with a distance of 20 m. Five soil cores were taken per plot to a depth of 20 cm using a stainless-steel soil auger with a 2.5 cm inner diameter and combined as one soil sample. Each soil sample was immediately frozen in liquid nitrogen and stored at −20 °C for DNA extraction.

### 2.2. DNA Extraction and Illumina HiSeq Sequencing

Microbial DNA was extracted from each sample using the E.Z.N.A.^®^ soil DNA Kit (Omega Bio-Tek, Norcross, GA, USA), and used for shotgun metagenomic sequencing at Biozeron Bio-technology Co., Ltd. (Shanghai, China). For each sample, 1 ug of genomic DNA was used with Illumina’s TruSeq for library preparation. Sequencing libraries were constructed using the Illumina HiSeq 4000, 150-bp paired-end technology. High-quality reads were assembled using Megahit [16], and the open reading frames (ORFs) of assembled contigs were generated to nonredundant unigenes after clustering using CD-HIT [17]. The generated Unique-gene set was searched against the NCycDB database [18] and NR database to retrieve N-functional gene and taxonomic annotations, respectively. All metagenomic data obtained in this study have been deposited in NCBI Sequence Read Archive (SRA) database under the accession number PRJNA730325. The 19 publicly available metagenomic data of marine samples (including marine sediments and seawater) were retrieved from the NCBI SRA database. A detailed description of the samples can be found in the previous study [19]. The metagenomic data from marine samples were processed in exactly the same way as the soil metagenomic data.

### 2.3. Statistical Analyses

All statistical analyses were executed using R (v4.0.2; https://www.r-project.org [accessed on 3 March 2024]) in Rstudio (https://www.rstudio.com/ [accessed on 3 March 2024]). The species detected in more than one of the soil samples and found to have one or more genes in a N-pathway were recognized as functional microbes of the N-pathway (i.e., the species could encode the N-pathway). The species was considered to possess N-metabolic versatility if it encoded two or more N-pathways. The Upset diagram was generated by using the “UpSetR” library [20]. Some species were randomly selected as representatives and visualized in a network using Cytoscape 3.7.0 software [21]. Heatmap showing the N-pathways the nitrifiers and N-fixers could or could not encode was rendered using an online tool (https://www.chiplot.online [accessed on 3 March 2024]).

## 3. Results and Discussion

### 3.1. N-Metabolic Versatility of Microorganisms in Soils

**Figure 1:**
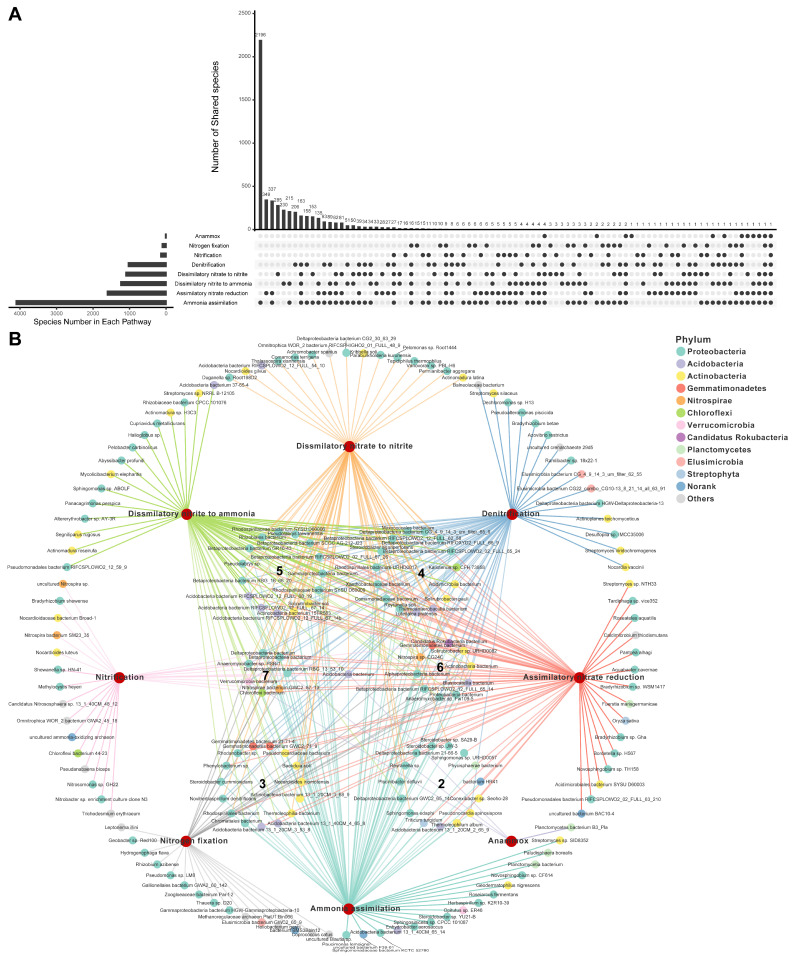
Microbes encoding one or multiple N pathways. (**A**) Upset diagram showing numbers of microbes (at the species level) encoding one or multiple N pathways. (**B**) Network displaying the species encoding specific N pathways. Only a random subset of species is shown here, please see Table S2 for a complete list of species and their involvement in N-pathways. The numbers in the middle of circles were the number of pathways a species could encode.

Across all soil samples, a total of 5363 species, belonging to 1144 genera, were detected to harbor N transformation genes and therefore were identified as N-transforming microbes (Figure 1). Among them, 2065 species belonging to 532 genera encoded more than one N-pathway (namely, possessing N-metabolic versatility), accounting for 38% of the total N-transforming microbes at the species level. Specifically, the number of species encoding two to eight N-pathways is 1021, 490, 292, 201, 46, 14, and 1, respectively. Among all the microbes with N-metabolic versatility trait, species with the ability to encode both ammonia assimilation and assimilatory nitrate reduction were the most numerous (337 species, 16%), followed by those with genes to carry out both ammonia assimilation and dissimilatory nitrite to ammonia (215 species, 10%) (Figure 1A). In terms of quantity, Actinobacteria, Alphaproteobacteria, and Betaproteobacteria were the three dominant taxa in microbes with N-metabolic versatility (Figure 1B), with 24%, 15%, and 14% species belonging to these three phyla, respectively. Although some N-transforming species are capable of participating in two N-transformation processes [13,22], it is unknown if N-metabolic versatility is a common trait of N-transforming microbes or only exists in a few special microbes. Our results showed that 38% and 53% of the individual N-transforming microbes, at the species and genus levels, respectively, were capable of participating in more than one N transformation pathway (Figure 1A), indicating that N-metabolic versatility is a common trait of N-transforming microbes.

**Figure 2:**
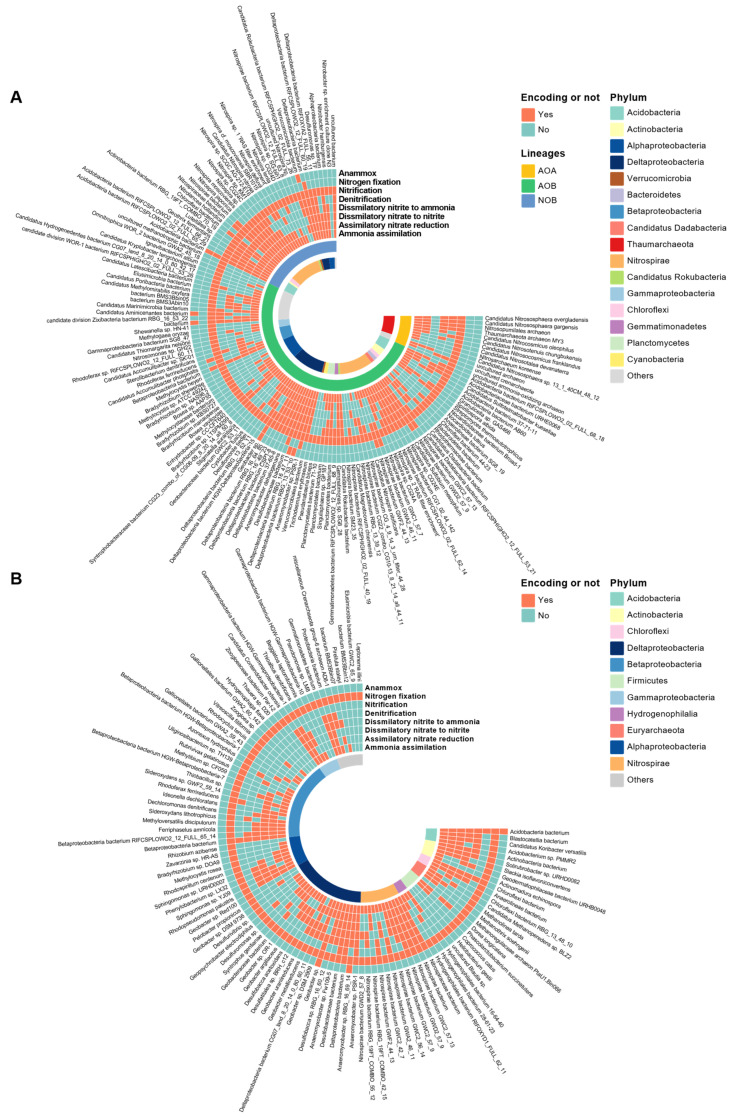
Heatmap showing the N-pathways the nitrifiers (**A**) and N-fixers (**B**) could or could not encode. The red color indicates the species could encode the N-pathway, while the blue represents could not. The affiliation of species at the phylum or class levels is indicated by different colors on the internal ring, phyla with less than 2 species are grouped into “Other”. See Table S2 for the versatility of the functional species responsible for remaining N-pathways.

The nitrifying and N-fixing microorganisms were used as examples to illustrate the N-metabolic versatility of N-transforming microorganisms (Figure 2). A total of 147 species containing one or more of the following genes: ammonia monooxygenase gene (*amoCAB*), methane monooxygenase gene (*pmoCAB*), hydroxylamine oxidoreductase gene (*hao*) and nitrite oxidoreductase gene (*nxrAB*), were detected and identified as nitrifying microorganisms (Figure 2A). Among them, 13 species of ammonia-oxidizing archaea (AOA, carrying the *amo* gene), 99 species of ammonia-oxidizing bacteria (AOB, carrying the *amo* and/or *hao*), and 35 nitrite-oxidizing bacteria (NOB carrying the *nxr*) were found. Among the 13 AOA species, 2 pure AOA species (i.e., carrying out nitrification merely) were found, accounting for 15% of the total number of AOA species. The remaining 85% of AOA species exhibited N-metabolic versatility (i.e., participating in two or more N-pathways). The largest number of AOA (4 species, or 31% of all AOA species) had an ammonia oxidation function accompanied by ammonia assimilation, assimilatory nitrate reduction, dissimilatory nitrate to nitrite, dissimilatory nitrite to ammonia, and denitrification function. Among the 99 AOB species, 12 species possessed only the *amo* gene, and the rest were armed with N-metabolic versatility, with the function of ammonia oxidation accompanied by ammonia assimilation, assimilatory nitrate reduction, dissimilatory nitrate to nitrite, dissimilatory nitrite to ammonia, and denitrification (14 species, 14% of the total AOB). Among the 35 NOB species, two only had the *nxr* gene, while the remaining NOB (94%) showed N-metabolic versatility, with 38% of them carrying genes related to ammonia assimilation, assimilatory nitrate reduction, dissimilatory nitrate to nitrite, dissimilatory nitrite to ammonia, and denitrification. Pure culture methods revealed that NOB are N-metabolically diverse as well, such as *Nitrospira moscoviensis* (a species also detected in this study) which is capable of decomposing urea to produce ammonium (i.e., involved in the ammonia assimilation process) [15], and most NOBs harbor *nirK* genes for the reduction of nitrite to nitric oxide, and some NOBs within the genus *Nitrospira* even participate in denitrification [15,23]. Our results showed that 85% of nitrifying microorganisms (at the species level) possessed N-metabolic versatility, and the species carrying out nitrification along with ammonia assimilation, assimilatory nitrate reduction, dissimilatory nitrate to nitrite, dissimilatory nitrite to ammonia, and denitrification were the most prevalent.

In this study, a total of 106 species with the N-fixing gene *nif* were identified as N-fixing microbes (Figure 2B). Among them, 15 species (14%) had only N-fixation genes, and the remaining 86% of N-fixers were equipped with N-metabolic versatility. The species having N-fixation capability along with ammonia assimilation, assimilatory nitrate reduction, dissimilatory nitrate to nitrite, dissimilatory nitrite to ammonia, and denitrification functions were the highest (16), followed by species with N-fixation and ammonia assimilation functions (10), accounting for 15% and 9% of the total number of N-fixing species, respectively. In this study, *Anaeromyxobacter* (belonging to the Deltaproteobacteria), a genus widely distributed in agricultural soils, possessed functional genes for ammonia assimilation, assimilatory nitrate reduction, dissimilatory nitrate to nitrite, dissimilatory nitrite to ammonia, denitrification, and nitrogen fixation. This result is in accordance with prior studies where the genus *Anaeromyxobacter* are functional microbes for ammonification [24], dissimilatory nitrate reduction to ammonium (DNRA) [10,25], denitrification (N_2_O to N_2_) [25], dissimilatory nitrate to nitrite (carrying narG and napA) [26], and N-fixation [27].

### 3.2. N-Metabolic Versatility of Microorganisms in Marine Ecosystems

The N-transforming species were also recovered from the publicly available metagenomic data of marine samples (including marine sediments and seawater). Marine N-transforming species differed greatly from those in soils, with 73% of the total 9117 species in marine not detected in soils (Figure S1). Although the N-transforming microorganisms responsible for N-cycling in marine and soil ecosystems were different, N-metabolic versatility was found as a common trait of these N-transforming microbes in both ecosystems. The marine microbes with N-metabolic versatility accounted for 35% and 51% of the total N-transforming microbes at the species and genus levels, respectively (Figure S2). Specifically, the number of marine species encoding two to eight N-pathways is 1630, 725, 444, 291, 90, 14, and 8, respectively. Unlike in soil ecosystems, species with N-metabolic versatility in marine ecosystems mainly belong to Alphaproteobacteria, Gammaproteobacteria and Bacteroidetes (Table S3), with 28%, 23%, and 9% of species belonging to these three phyla, respectively. Furthermore, more than 83% and 88% of the marine nitrifying and N-fixing microbes were N-metabolic versatile, respectively. Our results indicated that N-fixing microbes in marine ecosystems mainly belong to Alphaproteobacteria and Cyanobacteria, which was consistent with previous studies that found Alphaproteobacteria also possess *nif* genes [28,29]. Although functional and taxonomic annotation of short-read sequences may be sparse and/or occasionally erroneous due to limitations of genomic databases, our analyses of overall N-cycling microbes suggest that N-metabolic versatility is a common functional trait of N-transforming microbes. The novel N-processes (e.g., nitrifier denitrification) and lineages identified with N-metabolic versatility deepen our understanding of N-cyclers [22,30,31]. For example, *Nitrospira* is traditionally defined as a nitrite oxidizer, but some of them can grow not only on nitrite but also ammonia and nitrate [32,33], and encode highly similar forms of nitrite oxidoreductase with anaerobic ammonium-oxidizing *planctomycetes* [34]. Our findings suggested that N-transforming microbes were more N-metabolically versatile than previously known.

## 4. Conclusions

In summary, more than 38% and 35% of N-transforming species in soil and marine ecosystems, respectively, possessed N-metabolic versatility, despite the significant differences in N-transforming species between them. Our results indicated that N-metabolic versatility is a prevalent characteristic among N-transforming microorganisms, which could expand our comprehension of their functional traits.

## Data Availability

All metagenomic data obtained in this study have been deposited in NCBI Sequence Read Archive (SRA) database under the accession number PRJNA730325.

## Supplementary Materials

The following supporting information can be downloaded at: https://www.mdpi.com/article/10.3390/microorganisms12071283/s1, Figure S1: Venn diagrams of N-transforming species’ distribution between soil and marine ecosystems; Figure S2: Upset diagram showing numbers of marine microbes at the species level encoding one or multiple N pathways; Table S1: Basic statistical information of each sample; Table S2: Nitrogen-transforming species in soils and their encoding N-pathways; Table S3: Nitrogen-transforming species in marine ecosystem and their encoding N-pathways.

## References

[1] Shen D., Qian H., Liu Y., Zhao S., Luo X. (2022). Nitrifier community assembly and species co-existence in forest and meadow soils across four sites in a temperate to tropical region. Appl. Soil Ecol..

[2] Meng X.T., Liao H.K., Fan H.X., Zhang X.C., Li Y.Y., Yao H.Y., Razavi B.S. (2021). The geographical scale dependence of diazotroph assembly and activity: Effect of a decade fertilization. Geoderma.

[3] Feng M., Adams J.M., Fan K., Shi Y., Sun R., Wang D., Guo X., Chu H. (2018). Long-term fertilization influences community assembly processes of soil diazotrophs. Soil Biol. Biochem..

[4] Li S., Luo Z., Ji G. (2018). Seasonal function succession and biogeographic zonation of assimilatory and dissimilatory nitrate-reducing bacterioplankton. Sci. Total Environ..

[5] Yang Y. (2021). Emerging patterns of microbial functional traits. Trends Microbiol..

[6] Mouillot D., Graham N.A.J., Villéger S., Mason N.W.H., Bellwood D.R. (2013). A functional approach reveals community responses to disturbances. Trends Ecol. Evol..

[7] Martiny J.B.H., Jones S.E., Lennon J.T., Martiny A.C. (2015). Microbiomes in light of traits: A phylogenetic perspective. Science.

[8] Escalas A., Hale L., Voordeckers J.W., Yang Y.F., Firestone M.K., Alvarez-Cohen L., Zhou J.Z. (2019). Microbial functional diversity: From concepts to applications. Ecol. Evol..

[9] Green J.L., Bohannan B.J.M., Whitaker R.J. (2008). Microbial biogeography: From taxonomy to traits. Science.

[10] Nelson M.B., Martiny A.C., Martiny J.B. (2016). Global biogeography of microbial nitrogen-cycling traits in soil. Proc. Natl. Acad. Sci. USA.

[11] Kuypers M.M., Marchant H.K., Kartal B. (2018). The microbial nitrogen-cycling network. Nat. Rev. Microbiol..

[12] Stein L.Y., Klotz M.G. (2016). The nitrogen cycle. Curr. Biol..

[13] Chan Y.-K. (1985). Denitrification by a diazotrophic Pseudomonas species. Can. J. Microbiol..

[14] Itakura M., Uchida Y., Akiyama H., Hoshino Y.T., Shimomura Y., Morimoto S., Tago K., Wang Y., Hayakawa C., Uetake Y. (2013). Mitigation of nitrous oxide emissions from soils by *Bradyrhizobium japonicum* inoculation. Nat. Clim. Change.

[15] Koch H., Lücker S., Albertsen M., Kitzinger K., Herbold C., Spieck E., Nielsen P.H., Wagner M., Daims H. (2015). Expanded metabolic versatility of ubiquitous nitrite-oxidizing bacteria from the genus Nitrospira. Proc. Natl. Acad. Sci. USA.

[16] Li D., Liu C., Luo R., Sadakane K., Lam T.-W. (2015). MEGAHIT: An ultra-fast single-node solution for large and complex metagenomics assembly via succinct de Bruijn graph. Bioinformatics.

[17] Fu L., Niu B., Zhu Z., Wu S., Li W. (2012). CD-HIT: Accelerated for clustering the next-generation sequencing data. Bioinformatics.

[18] Tu Q., Lin L., Cheng L., Deng Y., He Z. (2019). NCycDB: A curated integrative database for fast and accurate metagenomic profiling of nitrogen cycling genes. Bioinformatics.

[19] Jian H., Yi Y., Wang J., Hao Y., Zhang M., Wang S., Meng C., Zhang Y., Jing H., Wang Y. (2021). Diversity and distribution of viruses inhabiting the deepest ocean on Earth. ISME J..

[20] Conway J.R., Lex A., Gehlenborg N. (2017). UpSetR: An R package for the visualization of intersecting sets and their properties. Bioinformatics.

[21] Shannon P., Markiel A., Ozier O., Baliga N.S., Wang J.T., Ramage D., Amin N., Schwikowski B., Ideker T. (2003). Cytoscape: A software environment for integrated models of biomolecular interaction networks. Genome Res..

[22] Zhu X., Burger M., Doane T.A., Horwath W.R. (2013). Ammonia oxidation pathways and nitrifier denitrification are significant sources of N_2_O and NO under low oxygen availability. Proc. Natl. Acad. Sci. USA.

[23] Park S.-J., Andrei A.-Ş., Bulzu P.-A., Kavagutti Vinicius S., Ghai R., Mosier Annika C. (2020). Expanded diversity and metabolic versatility of marine nitrite-oxidizing bacteria revealed by cultivation- and genomics-based approaches. Appl. Environ. Microbiol..

[24] Hu X., Liu X., Qiao L., Zhang S., Su K., Qiu Z., Li X., Zhao Q., Yu C. (2021). Study on the spatial distribution of ureolytic microorganisms in farmland soil around tailings with different heavy metal pollution. Sci. Total Environ..

[25] Sanford R.A., Wagner D.D., Wu Q., Chee-Sanford J.C., Thomas S.H., Cruz-García C., Rodríguez G., Massol-Deyá A., Krishnani K.K., Ritalahti K.M. (2012). Unexpected nondenitrifier nitrous oxide reductase gene diversity and abundance in soils. Proc. Natl. Acad. Sci. USA.

[26] Huang S., Chen C., Jaffé P.R. (2018). Seasonal distribution of nitrifiers and denitrifiers in urban river sediments affected by agricultural activities. Sci. Total Environ..

[27] Fan K., Delgado-Baquerizo M., Guo X., Wang D., Wu Y., Zhu M., Yu W., Yao H., Zhu Y.-g., Chu H. (2019). Suppressed N fixation and diazotrophs after four decades of fertilization. Microbiome.

[28] Martínez-Pérez C., Mohr W., Schwedt A., Dürschlag J., Callbeck C.M., Schunck H., Dekaezemacker J., Buckner C.R.T., Lavik G., Fuchs B.M. (2018). Metabolic versatility of a novel N_2_-fixing Alphaproteobacterium isolated from a marine oxygen minimum zone. Environ. Microbiol..

[29] Koirala A., Brözel V.S. (2021). Phylogeny of nitrogenase structural and assembly components reveals new insights into the origin and distribution of nitrogen fixation across bacteria and archaea. Microorganisms.

[30] Santoro A.E. (2016). The do-it-all nitrifier. Science.

[31] Kraft B., Jehmlich N., Larsen M., Bristow L.A., Könneke M., Thamdrup B., Canfield D.E. (2022). Oxygen and nitrogen production by an ammonia-oxidizing archaeon. Science.

[32] Daims H., Lebedeva E.V., Pjevac P., Han P., Herbold C., Albertsen M., Jehmlich N., Palatinszky M., Vierheilig J., Bulaev A. (2015). Complete nitrification by Nitrospira bacteria. Nature.

[33] Füssel J., Lücker S., Yilmaz P., Nowka B., van Kessel M.A.H.J., Bourceau P., Hach P.F., Littmann S., Berg J., Spieck E. (2017). Adaptability as the key to success for the ubiquitous marine nitrite oxidizer Nitrococcus. Sci. Adv..

[34] Lücker S., Wagner M., Maixner F., Pelletier E., Koch H., Vacherie B., Rattei T., Damsté J.S.S., Spieck E., Le Paslier D. (2010). A Nitrospira metagenome illuminates the physiology and evolution of globally important nitrite-oxidizing bacteria. Proc. Natl. Acad. Sci. USA.

