# Supplementary material for: Quest for the Nitrogen-Metabolic Versatility of Microorganisms in Soil and Marine Ecosystems
Source: Microorganisms. 2024 Jun 25;12(7):1283. doi: 10.3390/microorganisms12071283 (PMC11278940; doi:10.3390/microorganisms12071283)
Supplement: Supplementary file 1 [file microorganisms-12-01283-s001.zip › Supplementary Information.pdf]

Supplementary Information

Quest for the Nitrogen-Metabolic Versatility of Microorganisms in Soil and Marine Ecosystems

Yongpeng Zhao [1], Xia Zhu-Barker [2], Kai Cai [1], Shuling Wang [1], Alan L. Wright [3], and Xianjun Jiang [1,*]

[1] College of Resources and Environment, Southwest University, 2 Tiansheng Road, Beibei, Chongqing 400715, China
[2] Department of Soil Science, University of Wisconsin-Madison, Madison, WI 53709, USA
[3] Indian River Research & Education Center, University of Florida-IFAS, Fort Pierce, FL 34945, USA
* Correspondence: jiangxj@swu.edu.cn; Tel.: 86-23-68251249

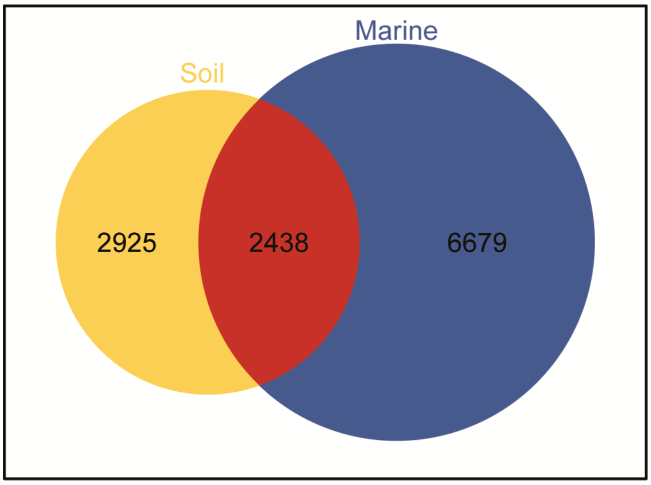

Figure S1. Venn diagrams of N-transforming species' distribution between soil and marine ecosystems. The number of species are indicated on different portion of each diagram. The size of each part is proportional to the number of species.

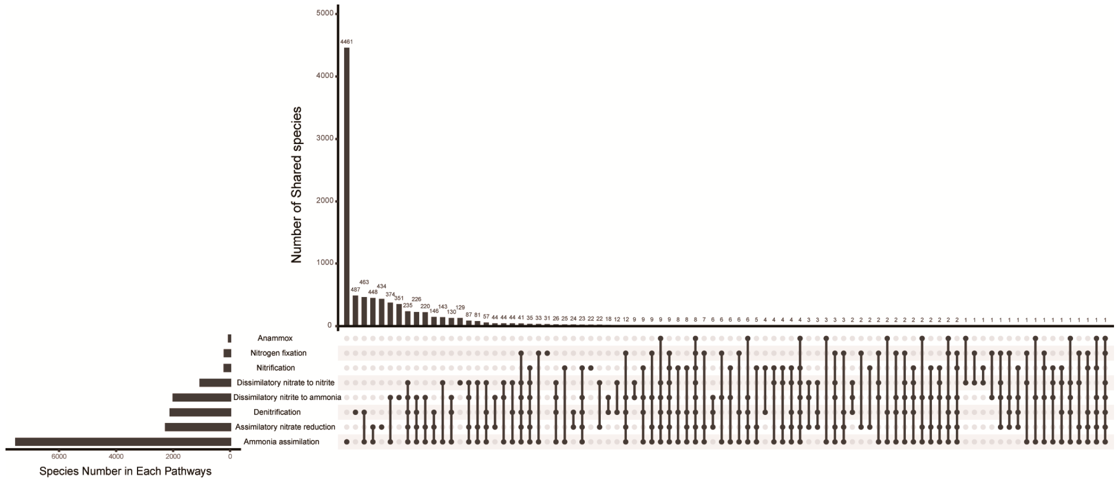

Figure S2. Upset diagram showing numbers of marine microbes at the species level encoding one or multiple N pathways.

Table S1 (xlsx). Basic statistical information of each sample.

Table S2 (xlsx). Nitrogen-transforming species in soils and their encoding N-pathways. “1” represents encoding the N-pathway and “0” means not.

Table S3 (xlsx). Nitrogen-transforming species in marine ecosystem and their encoding N-pathways. “1” represents encoding the N-pathway and “0” means not.
